# Nocebo effects by providing informed consent in shared decision making? Not necessarily: a randomized pilot-trial using an open-label placebo approach

**DOI:** 10.1186/s12910-020-00541-y

**Published:** 2020-10-14

**Authors:** Fabian Holzhüter, Johannes Hamann

**Affiliations:** Klinik und Poliklinik für Psychiatrie und Psychotherapie, Klinikum rechts der Isar, Technische Universität München, Möhlstraße 26, 81675 Munich, Germany

**Keywords:** Informed consent, Simple consent, Nocebo, Placebo, Shared decision making, Open-label placebo

## Abstract

**Background:**

Thorough information of the patient is an integral part of the process of shared decision making. We aimed to investigate if detailed information about medication may induce nocebo (or placebo) effects.

**Methods:**

We conducted a randomized, single-blind, pilot-study including *n* = 51 psychiatric in-patients aged between 18 and 80 years with a depressive disorder and accompanying sleeping disorders. In the intervention group we provided thorough information about adverse effects, while the control group received only a simple consent procedure. In both groups, patients received an open-label placebo pill instead of their sleeping medication.

**Results:**

No statistically significant differences between the intervention group and the control group were found regarding the main outcome parameter (a visual analogue scale indicating impairment by the new pill).

**Conclusion:**

In this study, we were not able detect an effect of informed consent vs. simple consent on the emergence of placebo or nocebo effects. This finding is contrary to most assumptions and publications about this topic.

**Trial registration:**

**Trial registration number:** DRKS00017653, registered August 30th 2018. Retrosprectively registered.

## Strengths and limitations of this study

- We performed an experimental randomized clinical trial with high clinical relevance.
- This study investigated a field of medicine that is lacking further research.
- There are weaknesses in the study design (e.g. a small number of participants).
- Open-label placebo is an under-researched topic in medicine [1]

## Background

Shared decision making (SDM) is a widely supported model of doctor-patient-interaction [2, 3] that may lead to benefits in patients’ clinical outcomes [4–6]. There is also an ethical imperative to overcome paternalistic structures and to involve patients in treatment decisions [7, 8].

In this study, we aimed to investigate whether detailed information about possible adverse effects of treatment options, as it is an obligatory step of SDM [9], may induce nocebo (or placebo) effects in patients suffering from depression (i.e. whether the amount of information given to the patient has positive or negative effects on the patient). Many physicians tend to avoid mentioning side effects and are reluctant because they fear to trouble the patient [10, 11]. This may hinder doctors to engage in SDM.

It has been postulated that placebo and nocebo effects may occur in the context of SDM [12, 13], still, to our best knowledge, there exist no clinical trials concerning this issue in mental health [1].

The aim of the present study was to investigate information provision as an important aspect of SDM with respect to potential nocebo effects.

## Methods

This study was a prospective, randomized, single-centered, subject-blind pilot study. We compared informed consent with simple consent about sleep medication for in-patients with depressive disorders. The Ethical Review Board of the Medical Faculty of the Technical University of Munich approved the study protocol (5708/13).

We recruited in-patients from one psychiatric university hospital in Germany and included female and male inpatients, aged between 18 and 80 years, diagnosed with a depressive disorder (ICD-10: F31, F32, F33, F41.2). Participants needed to be on medication for a documented sleep disorder.

For all patients, regular sleeping medication was paused for one night and replaced by a placebo pill in order to standardize drug effects between groups. Before obtaining written consent from the patients, we handed out an information sheet about our study. In this context, we explained to the patients that instead of their usual sleeping pill, they would receive a sham medication, known as placebo, which does not contain any active component. We also explained to the patients, that based on other scientific studies, placebos can have positive effects and that similar mechanisms are activated in the body as by regular medication. Thus, we performed an *open-label placebo* approach [14, 15]. Other studies found evidence that an open-label placebo administration was superior to no-treatment or treatment as usual [16, 17]. After patients filled out our baseline questionnaire, we used the phrasing “new sleeping pill” in order to remind the patients they’ll receive their placebo in the evening (see Table 1).

**Table 1:** Intervention and control condition

Control	Intervention
simple consent	informed consent
Dear Mrs. / Mr. [], just as we discussed earlier, you will receive a new sleeping pill for tonight.	Dear Mrs. / Mr. [], just as we discussed earlier, you will receive a new sleeping pill for tonight.
	Before that, I would like to give you some more information about that drug. The sleeping pill improves falling asleep and sleeping through the night in about 60% of all patients. It does not work for about 40% of all patients. No serious side effects are to be expected, but of course, like in all other medicaments, side effects can occur. About those, I would like to give you some more information: Around 30% of all patients report dry mouth. Around 27% of all patients report slight vertigo. About 40% of all patients report increased sweating.
As usual, you will be given that pill about half an hour before going to bed. Tomorrow, we will hand out a questionnaire to you, in which you can indicate how you have been sleeping tonight.	As usual, you will be given that pill about half an hour before going to bed. Tomorrow, we will hand out a questionnaire to you, in which you can indicate how you have been sleeping tonight.

We used a white placebo pill (7 mm) containing lactose monohydrate, cellulose powder, magnesium stearate and microcrystalline cellulose.

In the intervention group (informed consent) we gave detailed information on nonspecific side-effects that may be caused by open-label placebo (i.e. dryness of the mouth, vertigo and sweating, [18]), while in the control group (simple consent) we only informed the patients shortly about receiving a new sleeping pill (Table 1).

After enrolment and written consent, patients completed the first questionnaire. Right afterwards, patients were included to the intervention or control group, using a randomization list. From this moment on, the investigator was un-blinded and started the standardized informative conversation (see Table 1). In the same evening, patients received the placebo instead of their regular sleep medication. In the next morning, each patient had to fill out the second questionnaire.

At baseline, we obtained patients’ socio-demographics and asked for specifications about the sleeping disorder, such as the frequency of sleep disturbances in the last 7 days, whether the patient suffered from sleep-onset insomnia, sleep-maintenance insomnia or combined, the satisfaction with the sleep condition and the satisfaction with the treatment of the sleeping disorder.

The day after, patients provided a *detailed, subjective rating of the last night’s sleep* (Table 2) including a *visual analogue scale* (main outcome parameter) indicating how impaired they felt by their sleep medication.

**Table 2:** Outcomes

	intervention	control	*p*-value
“Please indicate how much you have felt impaired by side effects” (main outcome) (1 “not at all” – 10 “very much”)	1.5 (±1.4)	2.8 (±3.0)	*p* = 0.06
“How well did you sleep last night?” (1 “very bad” – 5 “very good”)	2.4 (±1.3)	2.1 (±1.2)	*p* = 0.56
“How much did the new drug cause side effects?” (1 “not at all” – 5 “a lot”)	1.3 (±0.7)	1.6 (±1.2)	*p* = 0.23
“How satisfied have you been with the new drug?” (1 “not satisfied at all” – 5 “very satisfied”)	2.0 (±1.3)	1.6 (±0.8)	*p* = 0.29
“How satisfied have you been with the amount of information about the new drug?” (1 “not satisfied at all” – 5 “very satisfied”)	4.5 (±1.1)	4.4 (±1.3)	*p* = 0.76
“How recreative was your sleep?” (1 “very recreative” – 6 “not recreative at all”)	4.2 (±1.7)	4.2 (±1.5)	*p* = 0.98
Time to fall asleep (min)	98.9 (±88.9)	108.0 (±87.4)	*p* = 0.55
Time spent awake during night (min)	115.9 (±113.2)	120.2 (±134.3)	*p* = 0.90
Time spent sleeping (min)	306.2 (±122.1)	335.0 (±157.0)	*p* = 0.47
Nightmares (Yes)	15.4% (*n* = 4)	16.0% (*n* = 4)	*p* = 1.00

The questionnaires were based on validated measures and additional specific questions. For the validated measures we referenced the respective literature [19–21]. A translation of the additional questions is attached.

Statistical analyses were conducted with SPSS® Version 24. Descriptive statistic included frequencies and means. For group comparisons we used t-test or *χ*^2^-test. A *p*-value < 0.05 was defined as statistically significant.

### Patient and public involvement

No patient involved.

## Results

Altogether 52 patients were recruited and data of 51 patients were analyzed (missing data for one patient). There were 26 patients in the intervention group and 25 patients in the control group. At baseline the two groups did not differ with regard to the patients’ age, gender, sleep quality and experience with medication.

The patients’ ratings concerning tolerability of the study medication and sleep quality showed no statistical differences between the groups for any outcome (Table 2). For the primary outcome (visual analogue scale indicating impairment by side effects) there was a statistical trend towards higher impairment in the control group.

## Discussion

Respectively, we were not able to show differences regarding side-effects and drug-efficacy between patients receiving simple and informed consent. Thus there was no hint towards nocebo (or placebo) effects deriving from the kind of information provision. The statistical tendency towards higher impairment in the control group opposed our presumption that a detailed information may induce side effects in patients.

### Limitations

The total number of 51 patients and the short duration of the intervention might have been too small to detect a possible difference between the two groups. During our conversation with the patient, we mentioned no severe or life-threatening side effects but only those that were likely to occur while taking a placebo pill. Also, the investigator informed the patients and the effects could be maximized by choosing another method or person.

Additionally, one could assume that the open-label placebo approach might have weakened possible effects and that an approach with deception might have maximized the findings [15]. Anyhow, we chose an open-label placebo approach for different reasons. Aiming to investigate how providing information may induce side effects, we saw no reason to deceive the patient and instead chose the most transparent study design. Since there are positive and negative results in literature for open-label placebo administration, there was no clear argument against it. In fact, our pilot study delivered results that showed no effect of this approach, which might be helpful for future studies.

Furthermore, the open label placebo approach had been highly recommended by our institutional review board.

Also, it is possible to object that the control group did obtain another kind of intervention instead of no treatment or treatment as usual, since all participants received the placebo pill and a short information in terms of simple consent.

## Conclusion

To conclude, we cannot support the assumption that nocebo effects can be induced by the extent of information provision to the patient. Still, there are weaknesses in our study design that may have skewed our findings. Unfortunately, and despite the importance of the topic, there is still a lack of studies focusing on the influences of doctor-patient-relationship on health outcomes. Thus, even though we could neither show a negative nor a positive effect of thoroughly informing the patient, further research is required to integrate our finding in an established concept. Especially as these findings oppose most publications that attribute nocebo effects to informed consent [22, 23]. Consequently, we want to encourage physicians to provide thorough information to their patients and not to be reluctant to take this important step of SDM. As implication for clinical practice, the way physicians discuss possible side effects with their patients is subject to an active discussion [24–26]. For example the concept of “contextualized informed consent” proposed by Kaptchuk et al. [27] appears to be a way of respecting patient autonomy and avoiding possible nocebo effects.

For further studies investigating this topic, we recommend measures to increase possible nocebo or placebo effects. Amongst others, this could be an administration with deception or by a person of authority. In addition, a greater study population might be necessary to detect differences between the groups.

## Supplementary information

**Additional file 1.** CONSORT 2010 Flow Diagram.

## Data Availability

There is data available concerning the results and questionnaires mentioned. The authors can access this data and it can be obtained from the authors upon reasonable request.

## Abbreviation

SDM: shared decision making
