# Supplementary material for: Nocebo effects by providing informed consent in shared decision making? Not necessarily: a randomized pilot-trial using an open-label placebo approach
Source: BMC Med Ethics. 2020 Oct 14;21:97. doi: 10.1186/s12910-020-00541-y (PMC7557071; doi:10.1186/s12910-020-00541-y)
Supplement: Supplementary file 1 — Additional file 1. CONSORT 2010 Flow Diagram. [file 12910_2020_541_MOESM1_ESM.doc]

**
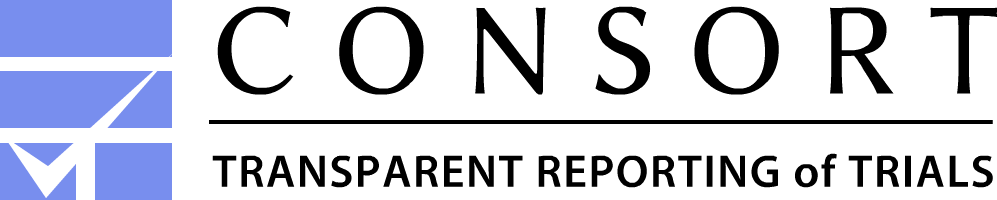
**

**CONSORT 2010 Flow Diagram**

**Allocation**

**Analysis**

**Follow-Up**

**Enrollment**

Assessed for eligibility (n=110)

Excluded (n=58)

♦  Not meeting inclusion criteria (n=49)

♦  Declined to participate (n=9)

♦  Other reasons (n=0)

Analysed (n=26)
♦ Excluded from analysis (n=0)

Lost to follow-up (give reasons) (n=0)

Discontinued intervention (n=0)

Allocated to intervention (n=26)

♦ Received allocated intervention (n=26)

♦ Did not receive allocated intervention (n=0)

Lost to follow-up (did not hand out questionnaire) (n=1)
Discontinued intervention (n=0)

Allocated to intervention (n=26)

♦ Received allocated intervention (n=26)

♦ Did not receive allocated intervention (n=0)

Analysed (n=25)
♦ Excluded from analysis (n=0)

Randomized (n=52)
